# Literature review on COVID-19 vaccine efficacy in the immunocompromised population, and possible implications to future vaccination in kidney transplant patients

**DOI:** 10.3389/fmed.2023.1103699

**Published:** 2023-02-02

**Authors:** Joo Kyung Park, Sunil Bhandari

**Affiliations:** ^1^Department of Renal Medicine, Hull University Teaching Hospitals NHS Trust, Kingston upon Hull, United Kingdom; ^2^Hull York Medical School, Kingston upon Hull, United Kingdom

**Keywords:** COVID-19, kidney transplant, hemodialysis, immunocompromised, vaccination, efficacy

## Abstract

Since the emergence of the virulent coronavirus in 2019, efforts to tackle the coronavirus-disease-2019 (COVID-19) pandemic have been made globally. The development of the coronavirus disease (COVID) vaccine was a significant breakthrough in ways to tackle the virus. Various research studies have been conducted to identify how the virus works and ways to manage COVID, including the efficacy of the vaccines. However, there is limited data on how these measures work for the immunocompromised, despite the grave impact of these virulent strains in this population. Specifically, this review aims to focus on kidney transplant recipients (KTRs). Studies have suggested that there is significantly lower vaccine response in some immunocompromised groups despite additional booster doses, and hence warrants an augmented or alternative protection against the severe acute respiratory syndrome coronavirus 2 (SARS-CoV-2) for these patients. This suggests a need for alternative or more tailored approach in providing adequate protection against the COVID-19 in these cohorts. Some suggested ways include withholding immunosuppressants before and/or after vaccination, increasing the vaccine doses or reducing intervals and providing a mixture of monoclonal antibody (mAb) or antiviral therapy. However, the appropriate degree of alteration and augmentation, as well as its safety and effectiveness remains to be determined. Furthermore, continuous emergence of more virulent strains, such as the Omicron and its sub-lineages or the Deltacron, emphasises the need for ongoing research to assess the effectiveness of the current treatment against these new variants. Overall, active interest and appropriate updates to COVID-19 guidelines is necessary.

## Introduction

In 2019, a highly transmissible and pathogenic version of coronavirus, severe acute respiratory syndrome coronavirus-2 (SARS-CoV-2), caused a global pandemic of Coronarvirus-Disease-2019 (COVID-19) that caused about 540 million confirmed cases and 6.3 million deaths (1). From immunocompromised to the general population (2) and from clinical to socio-economic implications (3, 4), COVID-19 had a significant effect on everyone. The emergence of such a severe and acute pandemic prompted a collaborative effort worldwide to tackle the virus, which included public health protection measures (isolation, social-distancing, hand-washing, mask wearing, intermittent lockdowns) and ultimately vaccinations. Several different COVID-19 vaccines have been developed from pharmaceutical companies at breakneck speed (4, 5), with many studies supporting the effectiveness of these vaccines in reducing mortality, disease severity and infection risk (5–10). However, there have been fewer studies comparatively that have examined vaccine efficacy in immunocompromised patients (2), despite the possible graver risks and consequences they face during this pandemic (11). Recent research demonstrates that the immunocompromised (such as those on immunosuppressive therapy or with a transplanted organ) may not achieve the same level of protection as the immunocompetent (2, 12–16). This review explores the current evidence for vaccine efficacy in the immunocompromised, specifically kidney transplant population, and its implications in respect to current COVID-19 preventative guidelines in the United Kingdom (UK).

## Vaccine response

A systemic meta-analysis reviewed 82 studies looking into seroconversion from the COVID-19 vaccine to compare its efficacy in the immunocompromised and the immunocompetent population (2). The majority of the included studies used messenger ribonucleic acid (mRNA) vaccines, although there were viral vector and inactivated whole virus vaccines included. The meta-analysis showed that organ transplant recipients were 16 times less likely to seroconvert after the first dose of a COVID-19 vaccine. Although a second dose improved seroconversion, it was of lower magnitude for organ transplant recipients, where only a third of the patients achieved seroconversion (2). The study suggested possible benefit of further vaccine doses (11), but recent data has shown persistently impaired spike protein response in a significant cohort of KTRs despite booster doses (12, 13). This poses the question of the benefit of additional doses in such cohorts, and may warrant the need of a guideline that appropriately advises additional protection methods against COVID-19 for this high risk group.

Focusing on kidney patients, the RECOVAC Immune-response Study, a multi-centre research in the Netherlands, demonstrated a significantly lower seroconversion rate in KTRs (14). The study analysed SARS-CoV-2 Spike S1-specific immunoglobulin G (IgG) antibody response, virus neutralising antibodies and SARS-CoV-2-specific T-cell response after two doses of mRNA COVID-19 vaccine in 800 participants of four different cohorts: 162 participants with chronic kidney disease (CKD) stage 4/5, 159 patients on dialysis, 288 KTRs and 191 controls. The data was collected over 4-month period in 2021 from February to May, at four different outpatient clinics of university medical centres in Netherlands. Data showed a seroconversion rate of 21.2% in KTRs, whereas 99% in controls, 96.3% in CKD stage 4/5, and 87.4% in dialysis cohorts. After the second dose, seroconversion rates improved to 56.9% in KTRs, although it was still much lower than 100% in the control group (14). Additionally, RECOVAC and several other studies further suggested older age, lower estimated glomerular filtration rate (eGFR), lower lymphocyte count, using mycophenolate mofetil/mycophenolic acid, not using steroids, and shorter time after transplantation to be associated with increased risk of being a non-responder (14–16). One study stated that lower lymphocyte count below 1.3 G/L associated with chronic immunosuppressive treatment was highly predictive of poor immune response in CKD patients (16). A single centre study with 707 KTR participants in the UK further suggested maintenance with mycophenolic acid to have the most negative impact on vaccine response (12). This again highlights the need for further research into these cohorts and possible additional measures to achieve a level of protection against COVID-19, including additional or alternative vaccination strategies (14–16).

The above mentioned studies and others point towards reduced vaccine response in allograft recipients after two (2, 15, 16) and even three (15, 17) doses of SARS-CoV-2 vaccine compared to the general population. However, some smaller sample studies showed possible benefit of further boosters (17–19). Both studies by Kamar et al. (19) and Alejo et al. (18) showed slightly improved humeral response after a fourth mRNA vaccine in patients with weak or no response after three doses. However, their sample sizes were 37 and 18 patients, respectively. Benotmane et al. (17) recruited 67 KTRs with low immune response after three mRNA vaccine doses and showed an improved serum neutralisation against the Delta variant, with an increase in proportion of patients having neutralising antibodies from 16 to 66% after the fourth vaccine. However, one third of the study sample remained unable to elicit serum neutralising antibodies. Despite the small sample size, this indicated that additional doses of COVID-19 vaccines may be beneficial in transplant patients even in previously weak or non-responders. To further support this, Osmanodja et al. (20) performed a retrospective study on 1,478 patients to look into dose adjustment regimens of mycophenolic acid on serological response against a fourth COVID vaccine. They concluded that up to five vaccine doses induced serological response effectively in KTRs, and that it could be further enhanced by pausing mycophenolic acid at the time of vaccination (except in those on belatacept therapy). This raises the question of potential benefit in higher or additional doses, prophylactic monoclonal anti-SARS-CoV-2 antibodies or even immunosuppressive modulation (17, 20).

Since the development of COVID vaccines, various SARS-CoV-2 variants have emerged, raising concerns of vaccine effectiveness against the newer dominant strains (21–23). Many of the studies were designed to assess effects of some combination of Moderna, AstraZeneca, or Pfizer vaccines, and they have shown levels of protection against the alpha, epsilon, beta, gamma etc., up to delta variants (17, 18, 21). More recent research aims to assess vaccine durability against the Omicron sub-lineages (22–24). Kirsebom et al. (22) conducted a test-negative case-control study that assessed vaccine efficacy against the Omicron in the UK, which included all vaccines used in England -Pfizer and/or Moderna. From January to March 2022, they accumulated 1,127,517 eligible symptomatic individuals’ test samples, of which 265,820 were BA.1 positive and 1,246,069 BA.2 positive, whilst 615,628 were negative controls. The vaccine effectiveness after the first dose was 14.8% against BA.1 and 27.8% against BA.2. This increased to 70.6% against BA.1 and 74.0% against BA.2 after booster doses, waning to 37.4 and 43.7%, respectively after 15 or more weeks post-booster dose. They concluded that there was no evidence of reduced vaccine effectiveness against symptomatic BA.2 disease compared to that of BA.1, and that there was no difference in rate of vaccine effectiveness decline as well. Although these results are consistent with neutralisation assays reported by the UK Health Security Agency (25), it differs from a Danish household transmission study that reported increased COVID infection susceptibility against BA.2 despite vaccination (26). Lyngse et al. (26) identified 17, 319 secondary infections in 22, 678 primary cases in a 1–7 day follow-up, concluding that the secondary attack rate was higher in Omicron BA.2 variant than that of BA.1, 39% and 29%, respectively. However, Kirsebom et al. (22) suggested that such discrepancy in the UK and Denmark studies may be due to differences in vaccination and previous infection history in these countries or due to methodology and study design, and that further research would be beneficial.

A single centre study from Ohio, United States (US), evaluated neutralising antibody titres against previous SARS-CoV-2 strains with D614G mutation, BA.3 and Deltacron (24). They sampled 10 vaccinated healthcare workers, 18 intensive care unit (ICU) patients 3 days post-admission regardless of vaccination status during Delta wave of the pandemic, and 31 hospitalised during the Omicron wave. The data was compared with prior reports on BA.1, BA.2 and Delta variants (24). In the healthcare worker cohort, neutralising antibody titres were 3.3 times as low against BA.3 and 44.7 times as low against the Deltacron variant. This proved to be 2.9 times as low in BA.3 and 13.3 times as low in Deltacron variants after a booster dose. In ICU patients, the titres against Deltacron were significantly lower –137.8 times as low as the titres against D614G variant. Interestingly, only 44.4% of the ICU cohort had titre levels above the limit of detection. The titre levels against D614G and BA.3 were similar. In the hospitalised during Omicron wave without ICU admission group, Deltacron and BA.3 neutralisation titres were similar, although much lower than data obtained during the Delta wave. Fully vaccinated healthcare workers with a booster dose had greater immune cover than the other cohorts during the Omicron wave, regardless of the vaccination status –titres 59.9 times higher than the patient cohorts. Evans et al. (24) concluded that the evolution of SARS-CoV-2 and possible emergence of more virulent variants are significant concerns that require attention.

The UK Medicines and Healthcare products Regulatory Agency approved the first bivalent COVID booster in late 2022 (27). This is a vaccine by Moderna that targets two COVID variants, hence containing 25 micrograms each of the vaccines targeting the original 2020 strain and the Omicron. It claims that exploratory analysis of the bivalent vaccine showed positive immune response against BA.4 and BA.5 Omicron sub-variants. This is supported from recent data which saw better neutralising activity in BA.5-containing bivalent booster against all Omicron variants including the newer BQ.1.1 and XBB strains compared to the monovalent booster cohorts (28). However, they noted generally reduced monovalent and bivalent vaccine neutralisation efficacy against the newer WA1/2020, XBB and BQ.1.1 strains (28). On the other hand, Wang et al. (29) reported significantly reduced neutralising capability of the mAb and vaccines against the newer BQ.1, BQ.1.1, XBB and XBB.1 subvariants, raising concerns on our current counter measures against COVID. Again, given the ongoing viral evolution, continuous research and attention to the immunocompromised population is crucial to optimally protect KTRs.

## Discussion

Despite additional booster doses, several studies suggest persistently weak or non-responders to the vaccine in the immunocompromised (13, 17, 18). Therefore, it may be beneficial for guidelines to routinely check vaccine response in KTRs after three to four vaccine doses to identify such cohorts, and to subsequently offer mAb or alternative methods of protection empirically to minimise severe disease. Although possibly expensive to realise, the potential cost savings of avoiding hospital admission and possible graft loss would be even greater, not to mention the impact on quality of life. A vaccination approach suggested by Caillard and Thaunat (13) proposes to incorporate routine spike antibody level check in order to provide sufficient protection against COVID. They recognised that specific follow-up with personalised intensified vaccination approach for KTRs is required, given numerous reports of only 4–48% of KTRs having detectable anti-spike IgG after the second vaccine. This approach considers measuring the anti-spike IgG levels after two doses of mRNA vaccine if no COVID history, and after the first vaccine with a previous positive COVID history. If spike antibody levels were low, it further suggests either considering additional booster doses, reducing mycophenolate mofetil dose and/or replacing it with belatacept, or providing primary prophylaxis with mAb based on the anti-spike IgG or spike-specific T-cell interferon-gamma responses. However, more data on the outcome of these recommendations are needed as, for instance, some studies showed a weak antibody response to three COVID vaccine doses in KTRs on belatacept (30).

There is still a lack of cumulative data to sufficiently advise a definite guideline. Although the presence of a cohort of weak or non-responders of vaccine have been identified, whether the failure to develop the anti-spike IgG implies continued SARS-CoV-2 susceptibility remains unclear (11, 31). Furthermore, the appropriate approach for enhanced COVID prevention is undetermined. Some suggest enhanced vaccination strategies, such as mixed dosing of vaccines with different mechanism of actions, alternative vaccination schedules, or even different dosing regimens (13, 31, 32). Others consider alternative strategies like mixture of mAb or altering immunosuppressive therapy (12, 33). Regardless, further studies are warranted to understand and establish the most appropriate approach to improve COVID-19 vaccine response and public health measures to protect the immunocompromised patients.

Currently in the UK, mAb are recommended as general COVID-19 treatment given that the patient meets certain criteria (34). There have been studies supporting positive therapeutic use of anti-spike mAb in KTRs (35–37) with mild COVID-19 infections as well. Ongoing research is looking into use of these neutralising mAb as preventative measures of COVID-19 in immunocompromised patients. A nationwide study in France compared COVID-related hospitalisation, 30-day ICU admission, and 30-day mortality rate in KTRs who received early mAb infusion and those who did not (37). Out of 235 KTRs, 80 patients received early mAb. The early mAb group had less COVID-related hospitalisation, ICU admission and 30-day mortality (35% vs. 49.7% in control), and no mechanical ventilation was required. This supported possible benefits of early administration of mAb in KTRs with mild COVID-19 *via* passive immunity.

Another interesting concept is using a mixture of mAb as a preventative and/or therapeutic management. For instance, REGN-COV2 is an antibody cocktail containing two mAb under ongoing double-blind study for its effect on reducing SARS-CoV-2 viral load, and its potential therapeutic and preventative use against COVID-19 (38). Their hypothesis is that COVID-19 mortality and complications are related to the viral load, and thus reducing the viral burden should result in clinical improvement. This was based on some recent data showing high viral titres in hospitalised patients (39), and increased mortality risk in higher viral loads (40). The *in vivo* studies in non-human primates showed significant reduction in viral load and enhanced viral clearance with REGN-COV2 in prophylactic and therapeutic contexts, respectively. In clinical context, their data showed enhanced viral clearance especially in patients with negative serum antibody, in other words whose endogenous immune response has not yet been initiated, or in those with high viral load at baseline. With patients who were already serum antibody positive, the exogenous mAb mixture did not improve immune response greatly, nor did it hinder the ongoing antiviral activity. This suggests prophylactic benefit in immunocompromised patients who did not have sufficient vaccine response to enhance protection against the virus.

Unfortunately, many clinical trials on mRNA COVID vaccines approved under Emergency Use Authorization in the US and Europe excluded patients on immunosuppressive therapy (33). However, there were reasonable suspicions of possible effects of immunosuppressants on vaccine efficacy. For instance, an American research group used a mouse model to demonstrate that immunosuppressive medications in autoimmune conditions may impair COVID-19 vaccine response (41). They treated mice with five different immunosuppressants: cyclophosphamide, leflunomide, methotrexate, methylprednisolone or mycophenolate mofetil, and compared the immune cell population pre- and post-immunisation with SARS-CoV-2 spike protein. All tested immunosuppressive therapies significantly reduced the serum antibody titres in the experimented mice.

Applying this to humans, a US case study of a 75 year-old male with myasthenia gravis under high-dose prednisone and mycophenolate reported no detection of SARS-CoV-2 neutralising antibodies after 4 weeks of the second mRNA vaccine (33). The patient received second series of Pfizer mRNA vaccination 42 days (first dose) and 63 days (second dose) following the last set of vaccines. But for the second series, he also had reduced dose of mycophenolate 3 weeks prior to the first vaccine and held both prednisone and mycophenolate the day before and three subsequent days after the second vaccine. No change in clinical status was noted throughout this observational study. Interestingly, neutralising antibodies were detected 2 weeks post-vaccination, suggesting improved immune response to COVID-19 vaccine after a pause in immunosuppressive therapy.

Specific to KTRs, the CPAT-ISR and BECAME trials are examples of prospective studies that aim to assess the effects of reduced immunosuppressive therapy in improving vaccine response in KTRs (42, 43). The CPAT-ISR is a US trial to assess COVID-antibody response to additional mRNA booster doses in kidney and liver transplant patients, either with or without immunosuppressant reduction (42). It will collect data from 15 different transplant centres across the US to observe if temporary dose reduction in immunosuppressants 5 days before and 2 weeks after booster doses will improve antibody response. Similarly, BECAME is a single-centre, investigator-initiated randomised controlled trial in Israel that will compare the SARS-Cov-2 seropositivity rate after third booster in patients with without reduced anti-metabolite (43). Although a smaller sample size compared to the CPAT-ISR, it will also look into T-cell response and perform an additional prospective observational study to include KTRs who received three vaccine doses, but were not eligible for the randomised controlled trial. Such trials will hopefully help identify how beneficial immunosuppressant modulation would be in improving vaccine response, and to establish a standard regime of approach on duration and dosing of reduction.

Given the nature of the rapidly evolving virus, studies and guidelines are being updated with possible need of holistic review and clarification. The UK Kidney Association provided a statement in December 2022 which recommended up to 6 doses of the vaccine in KTRs and patients with kidney disease on significant immunosuppressive therapy, and for offering COVID antibody testing for high-risk patients (44). They also acknowledged the need of clarification of mAb in CKD4/5 patients, in line with some recent studies suggesting clinical inefficacy of some mAb (amubarvimab, romlusevimab, sotrovimab, casirivimab-imdevimab, and amubarvimab-romlusevimab) and suggesting additional paxlovid (45). However, current regulations and recommendations remain to limit safe use of paxlovid or remdesivir in renal impairment patients (44). Evulsheld (Tixagevimab and casirivimab), currently under PROVENT phase III trial which includes CKD and immunocompromised patients, was given emergency use authorisation for the FDA (46), which may be a potential alternative and/or additional option for protection as suggested by the National Kidney Foundation (47).

## Conclusion

Continued global efforts to appropriately update our COVID management approach are essential to ensure protection of our immunocompromised patients. With less restrictive public regulations and newer studies suggesting a significant cohort of insufficient vaccine responders, guidelines should be revised/updated to reflect this. Routinely measuring vaccine response *via* spike antibodies and serum neutralisation, and thus offering mAb, alternate vaccine regimen or immunosuppression reduction as appropriate may be considered and studied. Efforts to promote vaccination and reduce vaccine hesitancy, and continued public precautions and care when visiting hospitals should be encouraged. Above all, further research is crucial to identify and establish appropriate measures, alongside active engagement from the government, healthcare and the public in order to continue to protect both the immunosuppressed and immunocompetent population.

## Author contributions

Both authors listed have made a substantial, direct, and intellectual contribution to the work, and approved it for publication.
